# Reproductive Performance of Mares Fed Dietary Zearalenone

**DOI:** 10.3389/fvets.2019.00423

**Published:** 2019-11-26

**Authors:** Carrie K. Vance, E. Heath King, Susan D. Bowers, Peter L. Ryan, Kevin Walters, Nancy W. Shappell

**Affiliations:** ^1^Department of Biochemistry, Molecular Biology, Entomology and Plant Pathology, Mississippi State University, Starkville, MS, United States; ^2^Department of Pathobiology and Population Medicine, Mississippi State University, Starkville, MS, United States; ^3^Animal Metabolism-Agricultural Chemicals Research Unit, Bioscience Research Laboratory, Edward T. Shaffer Agricultural Research Service, USDA, Fargo, ND, United States

**Keywords:** estradiol, E-screen, ovulation, pregnancy, progesterone, mycotoxin, horse

## Abstract

It is known that zearalenone (ZON) interacts directly with estrogen receptors, and its *in vivo* effects on reproduction have been well-documented in several species. In contrast, reports of ZON's impact on horse reproduction are conflicting and inconclusive, some studies confounded by the presence of mycotoxins such as deoxynivalenol in the feed. This study assesses the effect of chronic consumption of zearalenone on reproduction in cycling mares fed >95% pure ZON (0, 2, or 8 mg/da; *n* = 7 mares/treatment) for three estrous cycles, followed by artificial insemination, through 16 days of pregnancy. Animals were on ZON treatment for between 70 and 121 days (average 84) depending on individual cycle patterns. ZON-induced changes in serum concentration of estradiol (E_2_) and progesterone (P_4_), and total estrogenicity were measured using RIAs and the E-screen assay, respectively. Effects on reproductive physiology and pregnancy were monitored by ultrasound and clinical parameters. No significant changes were found in reproductive hormone levels of E_2_, or P_4_ for mares on ZON treatments compared to controls, although there was a significant (*P* < 0.01) increase in P_4_ levels across Cycle number in High ZON (8 mg/da) treated mares. There was also an increasing trend in the interovulatory interval in the High ZON treatment group. The overall estrogenicity was similar across treatments and over time, not differing from controls or between ZON treatment groups. Adverse uterine and ovarian effects were also not observed, but pregnancy rates were mixed with only 4 of 7 mares on Low ZON becoming pregnant, and only 3 maintaining pregnancy and fetal heartbeat by Day 30, compared to 5 of 6 control mares and all 7 mares on High ZON. Because reproductive efficiency and hormone concentrations are highly variable across individuals, this study did not demonstrate that ZON at 2 or 8 mg/da was detrimental to mares' reproduction. Yet, inferring that ZON treatments were completely without effect is also not appropriate, as the absence of measurable significant differences could be attributed to the limited sample size. Most importantly, there were no extreme signs of toxicology, in contrast to previous reports when ZON was fed at these “doses.”

## Introduction

Zearalenone (ZON) is one of several stable mycotoxins produced by *Fusarium* species which contaminates a variety of forages and cereals such as barley, corn, oats, sorghum, soy, and wheat (1, 2), and has been recently found in sugar beet pulp, a feedstuff often used as horse feed (3). Literature reports of hyperestrogenism, reproductive disruptions, and fetal abnormalities as a result of unintentional or intentional feeding of mycotoxin-contaminated feed have been recorded in numerous species (4–7). Often mycotoxin contaminated feed contains a mixture of compounds such as ZON, deoxynivalenol (DON), and T-2 toxins, among others, and deciphering the contribution of each individually or in synergism with each other is of interest (4). ZON and its derivatives α- and β-zearalenol (ZOL) are known to have affinity for estrogen receptors (8–10) and elicit a proliferative response in estrogen responsive tissues *in vitro* (11, 12). However, in studies that have assessed the adverse reproductive effects of administering ZON *in vivo*, comparison across livestock species demonstrates wide variations in both ZON metabolism and sensitivity, including that of ZON metabolites (5, 13–16).

The few existing reports of the *in vivo* effect of ZON on horses are confounded by some uncertainties about the role of ZON alone or in combination with other mycotoxins, and draw a range of conclusions. In an early study with controlled feeding of a mixture of ZON (2 ppm) + DON (20 ppm) mares exhibited reduced feed intake and interrupted estrous cycles (17). A few years later, a case study cited in most ZON reviews (5, 18, 19) reported contaminated feed (corn screenings) containing 2.7 ppm ZON was associated with mares (15 of 37) exhibiting signs of estrogenic toxicosis, including enlarged edematous vulvas and uteri, prolapsed uteri and internal hemorrhage, and severe flaccidity of genitalia in 2 of 11 stallions and some deaths (20). Horses were estimated to have consumed as much as 10.8 mg/da ZON in their daily intake (4 kg/da) of contaminated feed over 15 days, after which they began to refuse feed, became sick, and reduced their consumption to an estimated 1.3 mg/da ZON for another 15 days. Actual ZON concentrations reported here are questionable though, as the thin layer chromatography method used was defined as semi-quantitative (21). DON was not in the suite of mycotoxins assayed for by Gimeno and Quintanilla, although they reported that no other mycotoxins were present in the feed. Considering that later DON was found to have no significant effect on horses in a 40 day feeding trial at 36–44 mg/da (22), the effects observed seemed to be legitimately contributable to ZON contamination despite the omission of DON in the assay. However, toxicology of the horses in this 1983 case study was further complicated by the presence of *Bedsonia* (*Clamydia spp*.) infections and pneumonia and both mares and stallions described as sick, died within 7–8 h of respiratory paralysis and sudden blindness.

In more recent studies, significantly adverse physiological effects were not observed after feeding mares a dose of 7 mg/da of 95% purified ZON for 10 days during the second half of an estrous cycle (23) or in mares fed a mixture of ZON (3 mg/da) and DON (36 mg/da) contaminated oats over two discontinuous estrous cycles (24). While no significant deleterious effects on reproductive hormones, cycle length or uterine histology were found, both authors reported increases in the number of follicles observed after acute ZON exposure. In the later study an increase in the incidence of hemorrhagic corpora lutea and follicular hematomas, characterized by trabecular fluid filled areas and coagula, was also found and motivated these authors to suggest ZON acts directly on the ovaries and blood flow (24). Additionally, no knowledge exists on whether ZON influences pregnancy rates.

The conflicting information on horse susceptibility to ZON prompts a thorough assessment of the effects of this mycotoxin on reproductive efficiency in mares, especially over long periods and across numerous estrous cycles, as well as pregnancy rates. At present, there are no regulatory standards for zearalenone in feedstuffs in the United States, but European guidance limits ZON to 2 ppm = 2 mg/kg in cereal products (25). The objective of this study was to examine effects of ZON exposure in cycling mares fed ZON at a Low (2 mg/da) and High (8 mg/da) dose, and over the course of three estrous cycles and 16 days into pregnancy; thus testing reproductive responses to ZON at the European regulatory limit and at a 4-fold higher dose nearing that reported as having severe negative effects upon chronic exposure. These data will provide the feed industry and regulatory agencies *in vivo* data for both chronic and dose-dependent zearalenone effects on horse reproduction, an important first step in developing feeding guidelines and tolerance limits for zearalenone in the US.

## Materials and Methods

### Animals and Treatment

Zearalenone (ZON) was initially purchased (Fermentek, Jerusalem, Israel, 98% purity), and screened for 16 possible contaminating mycotoxins (<0.5 ppm) by the North Dakota State Veterinary Diagnostic Lab; and later generously provided by Merck Animal Health Laboratories. Zearalenone stocks were prepared in ethanol (4 and 16 mg/mL) and stored at −20°C. Stock ZON (0.5 mL) soaked into an apple wafer horse treat (MannaPro, Chesterfield, MO) was hand-fed daily to mares on ZON treatment in the morning (08:00), while control mares received an apple wafer containing 0.5 mL ethanol vehicle. Zearalenone doses of 2 mg/da (2 ppm) and 8 mg/da (8 ppm) were calculated based on a standard feed intake of 1.0 kg (2.2 lb) of sweet feed pellets by nose-bag (Freely's Equine Formula E2408AAA; ADM Alliance Nutrition, Inc. Quincy IL). Mares also had access to supplemental hay and pasture *ad libitum* (three round bales of hay/week of common Bermuda grass mix pasture, Mississippi State University).

Beginning between late spring and early summer, and prior to experimental treatment, 21 light horse mares were assessed for reproductive health; uterine biopsies were taken and mares were monitored for estrus by ultrasonography (US). Mares were biopsied at the base of either the right or left uterine horn. The endometrial tissue was then fixed in 10% formalin and histologic sections were examined by a boarded pathologist and graded using the Kenney-Doig Grading System (26) (Supplementary Table 1). Examination of all biopsies was done blind to the treatment class. Endometrial biopsies were performed instead of uterine culture because biopsy is the gold standard for diagnosing endometritis (27, 28). Uterine culture is used to detect bacterial contamination but when evaluated critically its sensitivity is lacking. In addition, uterine cultures are also easily contaminated and their interpretation could be misleading (28). Biopsies were also chosen to assess whether any changes in uterine architecture were observed in mares fed zearalenone. It is known that hyperestrogenism induces squamous metaplasia in tissues of other species (29).

Confirmation of estrus was defined as the presence of an ovarian follicle ≥ 32 mm followed by ovulation, at which point the day of ovulation was designated Day 0 of the estrous cycle and the beginning of ZON treatment. Blood was collected by venipuncture on Day 0 and concurrent physiological analysis of cervix size, uterine tone, and follicular consistency was monitored by palpation. Mare receptivity to a stallion was assessed using teasing codes and degree of uterine edema.

**Figure 1:**
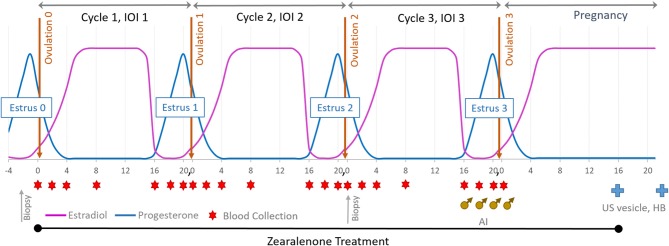
Experimental design: mares were exposed to ZON for three consecutive estrous cycles starting at Day 0 of the first cycle and through to Day 16 after breeding (AI). Blood was collected on Days 0, 2, 4, 8, and 16 and then daily until ovulation. Upon detection of a 32 mm follicle and uterine edema, serum was collected and reproductive activity examined daily until ovulation was confirmed. All mares were artificially inseminated on their 3rd estrus. US was used to determine pregnancy on Day 16 (vesicle) and Heartbeat (HB) on Day 30.

Balancing age, weight, and reproductive history, mares were assigned to one of three ZON treatment groups (7 mares/treatment): Control (0 mg/da), Low dose (2 mg/da), or High dose (8 mg/da), Table 1. Zearalenone treatments (Experimental design, Figure 1) began on the day of ovulation (Day 0) and continued daily for the following three consecutive estrous cycles and through Day 16 of the fourth cycle, after artificial insemination (AI). Treatment of horses was approved by Mississippi State University's Institutional Animal Care and Use Committee, protocol #12-022.

**Table 1:** Mare characteristics and experimental parameters.

**Mare characteristics at start of study**					**Zearalenone treatment**				**Average values of parameters**
**ID**	**Age (yrs)**	**Weight (kg)**	**Foaled**	**Biopsy grade**	**Start date**	**End date**	**Dose**	**# Days of treatment**	
923	3	464	N	1	May 24	July 19	Con, 0 ppm	62	
KW4	7	443	Y	2A	May 31	Aug 19	Con, 0 ppm	75	Age = 7.1
521	15	400	Y	2A	May 31	Aug 17	Con, 0 ppm	79	Weight = 471
601	12	511	Y	2A	June 4	Aug 18	Con, 0 ppm	75	
929^†^	*3*	*434*	*N*	*1*	-	-	*Con, 0 ppm*	-	Treatment
928	3	495	N	?	July 5	Oct 2	Con, 0 ppm	89	Days = 76.5
Precious	3	514	N	1	July 18	Oct 5	Con, 0 ppm	79	
814	13	392	Y	1	May 27	Aug 17	Low, 2 ppm	82	Age = 8.6
816	15	416	Y	1	May 30	Aug 17	Low, 2 ppm	79	Weight = 459
202	3	436	N	1	June 1	Aug 21	Low, 2 ppm	82	
KW2	7	420	Y	1	June 2	Aug 24	Low, 2 ppm	84	Treatment Days
206^*^	*3*	*530*	*N*	*1*	*June 7*	*Oct 6 PCL*	*Low, 2 ppm*	*121*	=^*^86.4 w/PCL
KW6	16	577	Y	2A	June 7	Sept 1	Low, 2 ppm	87	= 80.1 w/o PCL
925	3	441	N	1	June 18	Aug 27	Low, 2 ppm	70	
201	3	354	N	2A	May 22	Aug 14	High, 8 ppm	84	
511	14	406	Y	2A	May 25	Aug 17	High, 8 ppm	84	Age = 8.9
205	3	445	N	1	May 26	Aug 6	High, 8 ppm	72	Weight = 454
KW3	5	545	Y	1	June 2	Aug 19	High, 8 ppm	79	
KW1	16	454	Y	2A	June 2	Sept 2	High, 8 ppm	92	Treatment
448	7	484	Y	1	June 3	Aug 15	High, 8 ppm	74	Days = 81.6
KW5	14	493	Y	1	June 4	Aug 27	High, 8 ppm	86	

† *Control Mare 929 had a PCL in the process of determining her initial ovulation and thus her data was omitted from the study thereafter*.

* *Mare 206 (Low ZON) in estrous Cycle 2 developed a PCL in her left ovary which lasted for 76 days from June 30 to Sept 12 without clear ovulation. However, a new follicle developed in the right ovary on August 20 and ovulation occurred on Sept 20. Mare 206 was artificially inseminated Sept 13, 15, 17, and 19 prior to ovulation and became pregnant with fetal heartbeat detected at day 30 post-ovulation. ZON treatment was continued through the 84 days between the 2nd and 3rd ovulation events and until the 16th day of pregnancy for a total of 121 days on Treatment. Including mare 206, the average is 86.4 days on Low ZON treatment; excluding mare #206 the average is 80.1 days on Low ZON treatment*.

### Reproductive Physiology

Mares were weighed weekly and monitored over the entire treatment period for signs of toxicosis and oral lesions, and observed daily for signs of estrus by visual assessment of external genitalia. Reproductive activity was monitored every other day by ultrasound of the ovaries (to monitor follicular development) and reproductive tract for three full consecutive estrous cycles and into early pregnancy. At suspected estrus, rectal palpation was conducted to determine uterine tone, and transrectal ultrasonography used to determine uterine edema, presence of intrauterine fluid, follicle number, size, and consistency. After treatment began, ultrasound of ovaries was performed in each ovulatory cycle to determine if chronic ZON exposure affected dominant follicular size and structure or traits of follicular development. An additional biopsy of uterine tissue was performed at the beginning of estrus on the 2nd cycle after 30–35 days of ZON treatments, Figure 1. All mares were artificially inseminated with ≥500 million progressively motile sperm from a common stallion on their 3rd estrus, and AI was repeated every 48 h until ovulation was confirmed, Figure 1 (**♂**). Zearalenone treatment was terminated on Day 16 after Ovulation 3 and initiation of pregnancy, with ultrasound used to detect presence of an embryonic vesicle on Day 16. Maintenance of pregnancy was confirmed on Day 30 by visualization of an embryo with a heartbeat, Figure 1(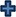).

### Serum Analysis

Blood samples (20 ml) were collected by venipuncture into 10-ml heparinized Vacutainer tubes starting on Day 0 when treatment was initiated and continued on Days 2, 4, 8, and 16, and then daily (when possible) until ovulation, through three subsequent ovulatory cycles, Figure 1 (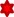). Serum samples were prepared on the day of draw, aliquoted sterilely and stored frozen at −80°C. Selected samples (Days 0, 2, 4, 8, and 16) were analyzed for estradiol (E_2_) and progesterone (P_4_) using ^125^I-radioimmunoassay Coat-a-Count® kits (Siemens Medical Solutions Diagnostics, Los Angeles, California) per directions. Assay sensitivity for E_2_ and P_4_ was 2.2 pg/mL and 0.025 ng/mL, respectively. Reported intra- and interassay coefficients of variation were 6.2% and 9.5% for E_2_ and 19.2% and 11.8% for P_4_ at the lowest concentrations, respectively. Duplicate analyses that differed by more than 15% were reanalyzed.

Total estrogenicity (estradiol equivalents, E_2_Eqs) was measured by the *in vitro* E-screen assay using the MCF-7 BOS, estrogen-dependent cell line (derived from a human mammary epithelial carcinoma, provided by Drs. Ana Soto and Carlos Sonnenschein, Tufts University, Boston, MA USA) as previously described (3, 30). Select serum samples (Days 0, 8) were extracted with acetonitrile (ACN 1:2 v/v), vortexed, centrifuged, and the serum/ACN supernatant dried and stored at −20°C. Sample pellet weights were used to calculate serum recovery. Extracts were resuspended in cell culture medium and dilutions tested to obtain a proliferative response in the linear range of the E_2_ standard curve. The limit of quantification was 0.02 pg/mL of E_2_Eq in the original serum.

### Statistical Analysis

All data are presented as Means ± SD. Estradiol and progesterone data were analyzed as a repeated measures design using the MIXED procedure of SAS (SAS Institute Inc.) and Analysis of Means using JMP 14.0.0 (Statistical Discovery^TM^; 2018 SAS Institute Inc.). Estrous cycle interval data was normalized to each mare's first cycle and analyzed. Normalization transforms did not reveal any additional features in analysis of means across treatments for any measured parameter. Sources of variation were ZON treatment and estrous cycle and their interactions. Means separations were performed using the Tukey-Kramer adjustment of multiple comparisons of least squares means and significance determined at the level of *P* < 0.05.

## Results

ZON-treated mares were assessed for impacts of chronic ZON exposure on reproductive function both in terms of clinical parameters and through hormone analysis. In late June, three mares were added to the control group when two mares were removed due to behavioral issues preventing data collection. During the study two mares exhibited persistent corpora lutea (PCL), one at the initial ovulation in the control group (#929, which had been added in late June), and one from the low ZON group (#206) after the second ovulation, resulting in an interovulatory interval (IOI) of 84 days. With the exception of mare #206, the length of ZON exposure ranged from 70 to 92 days (as a result of variable estrous cycle lengths for each individual) averaging 80.1 and 81.6 days for both Low and High ZON groups, respectively, Table 1. Final body weights were no less than 96% (Control group) of the initial weights, with group COVs ≤ 5% (coefficient of variation, data not shown). Teasing with a stallion was found an ineffective tool for detection of estrus, a consequence of mare inexperience and/or nature of individual mares (data not shown).

### Clinical and Physiological Analysis

The first estrous cycles for all ZON-treated mares occurred between May 21 and June 18. The three replacement Control mares, #929, 928, and Precious, were added on June 13, July 5, and July 18, respectively, Table 1. While mare #929 was removed due to a PCL on her initial cycle, Mares #928 and Precious were in the study until early October, while all other mares completed three estrous cycles and were artificially inseminated before or around the end of August. Thus, these two late added mares in the control group had their last estrus and were artificially inseminated late in the natural breeding season at the beginning of October and near the fall transition (31).

**Figure 2:**
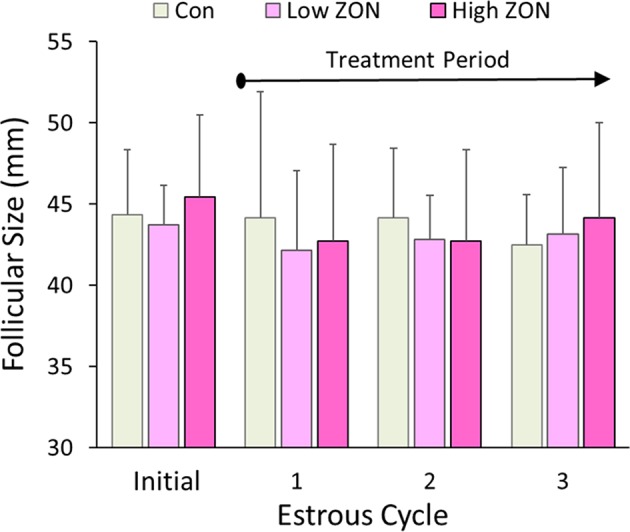
Dominant follicle size at ovulation within each of three estrous cycles with chronic ZON exposure compared to pre-exposure follicle size in cycle 0. Values are Means ± S.D.

Dominant follicular size measured by US prior to ZON/Control treatment was 44.5 ± 0.86 mm for all 20 mares in the study (excluding mare #929 due to PCL). Average dominant follicular size in each of the three estrous cycles is compared to the follicular size in the initial estrous across all treatments in Figure 2. Follicle sizes ranged from 42 to 44 mm, and no differences were observed in follicle size across estrous Cycles or ZON treatment.

The initial uterine biopsies taken just before Ovulation 0 (Table 1), showed scores of 2A in biopsies from 6 of 8 older mares (≥7 years of age), and 1 of 7 younger mares (<7 years old). The remaining mares had initial biopsy scores of 1 (biopsy notes available upon request). After 30–35 days of ZON treatment, biopsy scores of two mares in the Low ZON group changed from 1 to 2A, as did scores of two mares from the High ZON group. Edema was noted in more of the later biopsies across all treatment groups, but no obvious treatment effects were observed. The only biopsy that scored as 2B came from a 14 year old mare after treatment with High ZON for 35 days.

**Figure 3:**
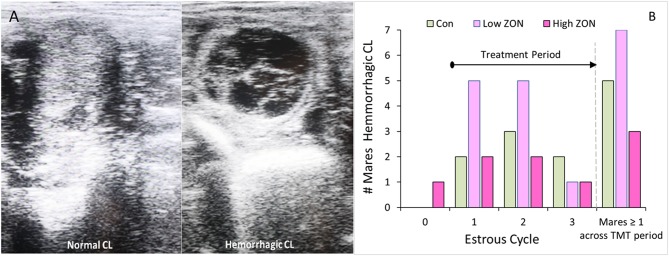
Corpus luteum development: **(A)** Ultrasound comparison of Normal and Hemorrhagic CL; **(B)** Incidence of hemorrhagic corpora lutea (HCL) by cycle, such that time on ZON treatment increases with cycle number. The last set of bars: number of mares by treatment having at least one HCL over entire study period. ZON treatments are: Control (*n* = 6) 0 mg/da, Low (*n* = 7) 2 mg/da, High (*n* = 7) 8 mg/da.

Pre- and post-treatment corpus lutea (CL) were observed for persistence and morphology by ultrasound, Figure 3A. Persistent corpus lutea (PCL) are defined as CL's lasting approximately 2 months (32). In this study, persistent CLs were formed in 2 of 21 mares, or ~10%. Mares with PCL were both 3 years old, and although one of these mares (#206) continued in the study, her data is considered only where indicated. Data from mare #929 was not included in any further analyses, due to presence of the initial PCL and no subsequent ovulation over the study period.

While hemorrhagic CL (HCL) are found to occur “naturally” in mares, and are not considered pathologic (31, 33), their presentation was recorded, Figure 3B. The frequency of HCL occurrence in mares at the initial estrous cycle prior to ZON treatment was 5% (1 HCL/21 animals, including both PCL animals). Afterwards, HCLs occurred in 5 of 6 Control (83%), 7 of 7 Low ZON (100%), and 3 of 7 High ZON (43%) mares. Frequency of HCLs [calculated from the number of HCLs/(number of mares × 3 cycles)] were 38, 52, and 24% for control, Low ZON and High ZON treatments, respectively. The value for Low ZON treatment includes mare #206, who exhibited an HCL on the first estrous cycle, and a PCL on the second estrous cycle, followed by a third “normal” estrous cycle. All animals in the Low ZON treatment group had HCLs at least once compared to only 43% of mares in the High ZON group. There was no relation between advanced age and HCL formation, and one 3-year old mare (#925) treated with Low ZON had HCLs in every estrous cycle. Both Control mares added later to the study had HCLs only on their “first” monitored cycle of the study, but this could have been their 2nd or 3rd estrous cycle of the season, as their first monitored cycle occurred in July.

**Figure 4:**
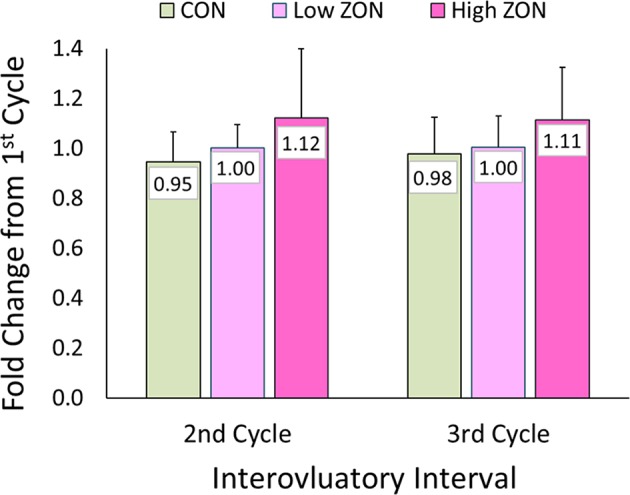
Change in interovulatory interval (IOI) with ZON treatment. Mean ratio ± S.D of each mare's 2nd and 3rd IOI to her initial IOI. Treatments: Control (*n* = 6), Low (*n* = 6), and High (*n* = 7). Low ZON treatment IOI does not include Mare #206 who exhibited a PCL in Cycle 2.

No statistical difference was found with ZON exposure on the interovulatory interval (IOI), Figure 4. Excluding mare (#206) with a persistent CL, the IOI ranged from 14 to 25 days. Because of the large variability of IOI among mares, data was normalized using each mare as her own control, with the ratio of the length of the second or third IOI compared to the length of the first IOI (Table 2 for each mare; Figure 4; Supplementary Figure 1, unadjusted IOI). The normalized mean IOI for control animals was 0.95 and 0.98 for the second and third cycles, respectively. Again, the two outlier mares with PCLs were omitted from this data set, as this would skew the data. Low ZON treated mares were similar to Control mares, exhibiting no IOI changes. However, the IOI for chronic High ZON exposed mares tended to be ~10% longer than the first IOI (though not statistically different, *P* = 0.23 for treatment effect) with group mean IOI of 1.12 and 1.11 for the second and third cycles, respectively. Within the High ZON treatment, 2 of 7 mares failed to have extended IOIs, Table 2. One mare had no change in IOI (100% and 96% of first IOI), while the other mare actually had much shorter IOIs (63% and 75% of first IOI, albeit her first estrous cycle lasted 24 days). Of the remaining 5 mares in the High ZON treatment, the increase in IOIs ranged from 10 to 50%. As found with hormone data below, individual animal responses were highly variable, even in the controls.

**Table 2:** Changes in interovulatory interval (IOI).

**ZON dose**	**IOI 2/IOI 1**	**IOI 3/IOI 1**	**MEAN change in IOI**
**RATIO of interovulatory intervals with chronic ZON treatment**			
	0.81	1.14	2nd/1st
	0.82	1.09	0.95 ± 0.05
Con	0.91	0.83	
	1.00	0.81	3rd/1st
	1.05	0.90	0.98 ± 0.06
	1.09	1.09	
	0.89	0.89	2nd/1st
	0.92	1.00	1.00 ± 0.04
Low	0.96	0.91	
	1.05	0.91	3rd/1st
	1.10	1.10	1.00 ± 0.05
	1.11	1.21	
	0.63	0.75	2nd/1st
	1.00	0.96	1.12 ± 0.10
	1.10	1.14	
High	1.10	1.20	3rd/1st
	1.20	1.20	1.11 ± 0.08
	1.33	1.11	
	1.50	1.43	

*Changes < 1.0 shaded in blue, >1.0 shaded in pink, darker shades reflecting ≥10% change*.

### Reproductive Hormones and Estrogenicity

**Figure 5:**
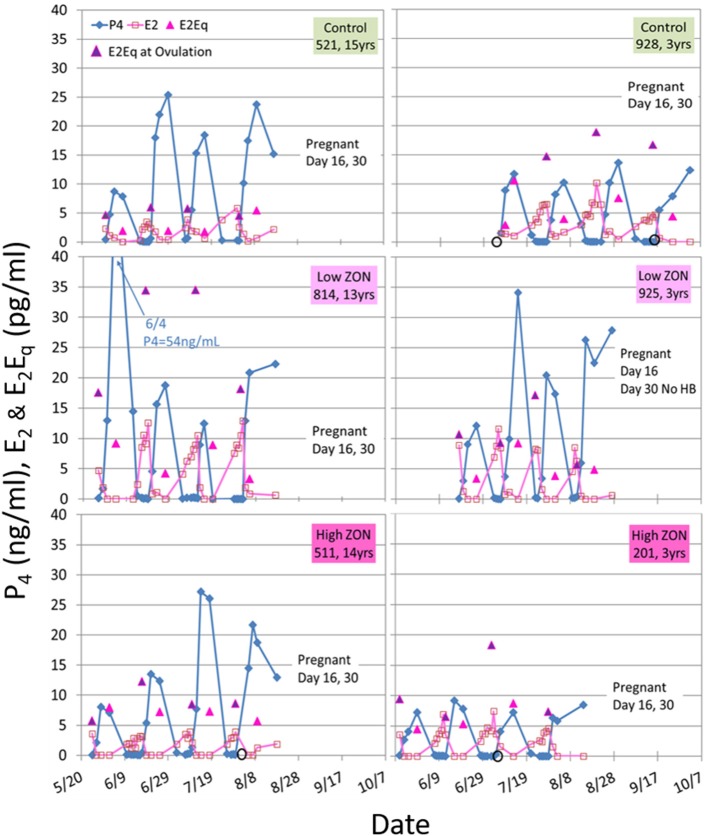
Estradiol (E_2_), progesterone (P_4_), and E_2_Eq values over three estrous cycles, and pregnancy status at the end of the 3rd ovulation after ZON treatment. The left side are older mares (13–15 years old) and the right side are 3-year old maidens. Estrogenic activity on the day of ovulation is indicated by the 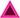 E_2_Eq. Ovulation is indicated by 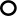 when missing a sample. Pregnancy was determined by ultrasound at Day 16, and fetal heartbeat at Day 30. The x-axis spans May 20 to October 7, as mares were started on the study on different dates based on their initial ovulatory events.

**Figure 6:**
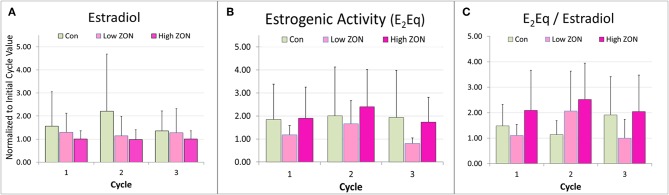
Peak **(A)** estradiol, **(B)** peak estrogenic activity, and **(C)** the ratio of E_2_Eq/E_2_ of Mares on ZON treatments. E_2_ and E_2_Eq were normalized to the mare's first cycle values. E_2_Eq/E_2_ was calculated as the ratio of the normalized values of E_2_Eq and E_2_ for each mare. Means ± S.D. are shown for three cycles.

Variability in hormone profiles of individual mares within treatment groups was present in all treatment groups, including the Control group. Hormone profiles for all mares are presented in Supplementary Figures 2A–C; two representative mares (one older, and one 3 year old) from each treatment group are shown in Figure 5. Estradiol was measured on days 0, 2, 4, 8, and 16 of each estrous cycle for all mares. Variability in hormonal response among mares was compensated for by normalizing values within each individual mare. Values of peak E_2_ were normalized for each mare relative to her values at Estrus 0 and group means of normalized values were compared, Figure 6A. Slight decreases in E_2_ concentrations compared to Control appeared with increasing ZON doses, most noticeably in Cycle 2.

Total estrogenicity in mare serum was measured using the E-screen assay which measures induced proliferation of MCF-7 cells via estrogen receptor binding by both endogenous estrogens and estrogen-like compounds, such as ZON. Interaction with the cell receptors and resultant cellular response should not be presumed to be additive, as competition between endogenous and exogenous compounds will occur (34). ZON treatment might be expected to increase overall estrogenicity of serum, yet no significant difference in ZON-treated mares compared to Control was seen (Figure 6B) and in fact E_2_Eq of Low ZON mares tended to be lower. The influence of the ZON treatment on the estrogenicity is clearer in Figure 6C when the ratio of the E_2_Eq to E_2_ is considered. In Cycle 2 the effect of ZON on estrogenicity is increasing while the endogenous E_2_ contribution is decreasing. However, in Cycle 3, the ZON contribution at the Low dose of 2 mg/da appears to diminish with longer exposure, although not significantly. By contrast, estradiol is nearly constant over time when mares were exposed to High ZON.

**Figure 7:**
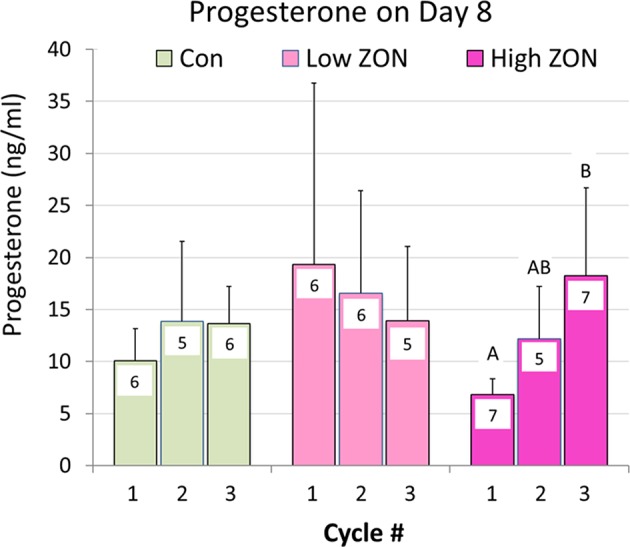
Progesterone collected on Day 8 of each Estrous Cycle. Means ± S.D. are shown for three cycles, with *n* indicated within the bar (variable due to some blood samples not collected). Low ZON treatment Cycle 1 includes one mare's P_4_ value that was 3.5X higher than the next highest mare. Progesterone Means for Cycles 1 and 3 of High ZON mares differed (*P* < 0.01).

Progesterone concentrations are presented in Figure 7. No overall differences were found in P_4_ levels on Day 8 of each estrous cycle of Low or High ZON treated mares compared to Control. However, a significant (*P* < 0.01) elevation in P_4_, was observed with chronic exposure of mares to High ZON over three estrous cycles. In the Low ZON treatment, early elevated values of P_4_ are largely contributable to the high variability in mares as one mare in Cycle 1 had a P_4_ value > 3X than the next highest mare in the group; analysis excluding this outlier decreases the P_4_ Means value from 19.3 to 10.0 ng/ml.

### Pregnancy

**Figure 8:**
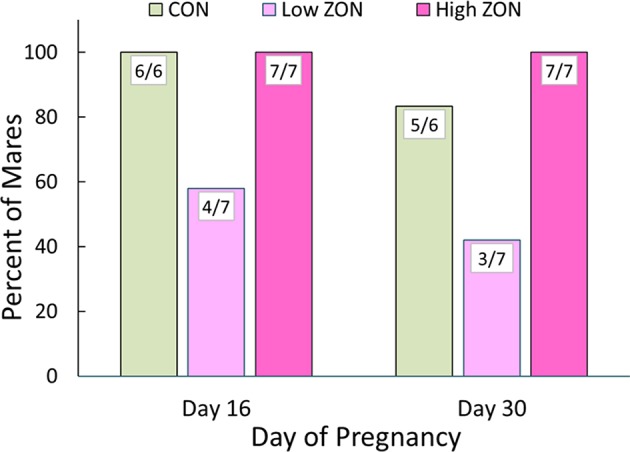
Incidence of pregnancy after chronic ZON treatment. Day 16 pregnancy confirmed by ultrasound detection of embryonic vesicle and Day 30 by heartbeat, Control (*n* = 6); Low (*n* = 7); High (*n* = 7). Mare #206 is included (121 days on Low ZON treatment, see text for details).

Ability to conceive was assessed by US detection of an embryonic vesicle at 16 days post-ovulation, and ZON treatments continued through Day 16. In common practice, human chorionic gonadotropin would typically be administered after insemination, but this was avoided to observe ZON's unmasked effects. Embryonic vesicles were found in all mares from the Control and High ZON treatment groups, while only 4 of 7 mares had embryonic vesicles in the Low ZON group (including mare #206), Figure 8. By 30 days post-ovulation, one Control mare and one Low ZON mare had failed to maintain pregnancy, assessed by lack of embryonic heartbeat, indicating spontaneous embryonic death, resulting in pregnancy rates of 83% and 43% in the Control and Low ZON treatment groups, respectively. All mares in the High ZON treatment group remained pregnant through Day 30.

## Discussion

The literature has multiple reports of the effects of ZON on reproduction in livestock [for review (6)], and often refers to the species sensitivity of pigs and other animals. However, the number of studies addressing the effect of ZON on reproduction in horses is extremely limited, consisting of two reports, in which the most alarming described a natural outbreak of ZON mycotoxicosis in 1983 (20), and three *in vivo* studies with control and treated mares (23, 24, 35) of which only one (Juhász) used only ZON, rather than a mixture of ZON and deoxynivalenone (DON) or a suite of toxins in contaminated feed. In the highly referenced natural outbreak of 1983, feed concentrations of ~2.7 ppm ZON were cited in connection with mares experiencing swollen vulvas, prolapsed vaginas, and edematous uteri as well as internal hemorrhage, the specifics and complications of this study were discussed in the introduction (20).

The difficulties in establishing deleterious effects of ZON on reproduction are complicated by the inherent variability in mare cycle length, and hormonal responses previously reported (36) which are further complicated by seasonal effects. In an attempt to overcome this variability, animals are often used as their own controls, as done by Aurich et al. (24), in which mares were observed during toxin-free feeding for 3 estrous cycles, before being exposed to mycotoxins for 2 alternating estrous cycles. Some of those findings were similar to our results; specifically, no statistical changes were observed in IOI and E_2_, although E_2_ peaks appeared to be slightly lower. Aurich et al. (24) reported luteinizing hormone concentrations, and these authors observed that peaks tended to be higher with toxin exposure. That study also found an increase in the number of small follicles (1–2 cm in diameter) during the later portion of the cycle, and presence of hemorrhagic CL (1.7 per mare, *P* < 0.01) and follicular hematomas (1.3 per mare). The occurrence of HCLs might be more related to the cycle number or season [reported by (31, 33, 37, 38)], as the highest incidence of HCLs occurred after the first and second estrus regardless of ZON exposure. In the current study, there also appeared to be a trend of decreased peak E_2_ serum concentrations with chronic feeding of 2 or 8 mg/da for three consecutive estrous cycles (Figure 6A). Additionally, while not statistically different, Low ZON tended to result in a higher frequency of HCL (1.6 per mare), and every mare in the study treatment group experienced at least one HCL. In another study, when 7 mg of pure ZON was fed as a bolus to mares for 10 days in the second half of a normal estrous cycle, no effect was observed on interovulatory interval, luteal and follicular phases, P_4_, or uterine edema (23). In the current study, while no statistical difference in IOI was observed, 5 out of 7 mares on High ZON treatment had increased IOI in both the second and third cycle of exposure. It was also observed that P_4_ increased significantly as mares were chronically exposed to 8 mg/ml ZON, which was not observed previously. The absence of a tendency for increased IOI in the mares reported by Aurich et al. (24) could have been the result of discontinuous ZON exposures (alternating cycles). Frequency of the incidence of persistent CLs (10%) was similar to the reported naturally occurring frequency of −10% in the peak of the ovulatory season (38, 39). There were no clear treatment effects on the biopsy results, and higher scores for older mares have been reported in the literature (40).

There appears to be some differential response in hormone levels when mares were treated with Low ZON vs. High ZON, Figures 5–7. Estradiol levels tend to be lower for mares subject to either ZON dose than for Control mares (Figure 6A), especially in Cycle 2. This may reflect decreasing endogenous E_2_ synthesis in an attempt by the mare's endocrine system to compensate for the estrogenicity of the exogenous mycoestrogen ZON. Yet estrogenic activity (a reflection of both natural estrogens as well as ZON and its metabolites, Figure 6B) tends to be lower primarily in Low ZON, especially in Cycle 3, but not in High ZON. The apparent decrease in E_2_ in the second cycle might be reflected as a decrease in serum estrogenicity, if E_2_ were the predominant driver of estrogenic activity, or it may reflect a higher clearance of the 2 mg/da dose of ZON vs. the 8 mg/da dose, resulting in E_2_Eq similar to values found in Control mares, despite lower E_2_ concentrations. Credibility to this theory is provided by an elegant metabolism study of ZON in mares (35) documenting an adaptive response to ZON exposure. Mares were fed ZON for 10 days at 2.7 mg/da, and plasma, urine, and fecal concentrations of ZON and its metabolites were measured on Day 1 (or Day 2 for feces) and Day 10. In that study, plasma ZON concentrations decreased by an order of magnitude by Day 10, but no change in α-ZOL was seen, and ß-ZOL increased by 8-fold. While urinary ZON and α-ZOL concentrations increased 4-fold, ß-ZOL increased 12-fold on Day 10, but fecal concentrations remained within ~70% of Day 2 values. These data show the capacity for the mare to adapt to ZON exposure, through increased clearance. Adaptation to ZON exposure was also indicated by another study in which horses of Italian origin were found to have lower concentrations of ZON and its metabolites in their urine than horses of Northeastern European origin. Yet when spermatozoa of the horses of Italian origin were exposed to ZON using an *in vitro* assay, they were found to be more sensitive to ZON toxicity (41). It could be inferred that because these horses had adapted to ZON exposure through decreased absorption and/or metabolism of ZON, no adaptive response had developed to minimize spermatozoa sensitivity to ZON.

It is unclear what the causative factor is of the rise in P_4_ concentrations in the High ZON treated mares. One possibility could be the inability to metabolically clear ZON at this dose, resulting in depressed E_2_ concentrations. This higher P_4_ concentration may be reflected in the trend of longer interovulatory intervals with expectations of longer lasting CLs, Figure 4. In addition, higher P_4_ concentrations may have contributed to the higher number of successful pregnancies, compared to the Low ZON mares.

Unique to this study is the question of ZON's effect on reproductive success. While the effects of ZON feeding at these levels on many parameters appear questionable at best, though not statistically testable, there would appear to be decreased fertility and reproductive success with Low ZON feeding. With only a 57% conception rate, and only 3 mares maintaining pregnancy through day 30, it seems unlikely that ZON was completely without effect. In fact, one mare on Low ZON that had maintained a persistent CL for 84 days after her 2nd ovulatory cycle, eventually ovulated, was successfully impregnated and maintained the pregnancy through day 30. This mare was chronically treated with 2 mg/da of ZON for nearly 121 days, and while under a normal commercial breeding scenario prostaglandin would have been administered to lyse the persistent CL, in this study the objective was to examine the direct effects of ZON without interference.

Finally, the real issue is to determine at what concentration, if any, is ZON a threat to a mare's reproductive performance. While regulatory agencies must base regulatory limits on the concentration of the toxic compound in the feed, the “dose” that any animal consumes is instead the critical factor in assessment of a toxin's potential for toxicity. The ZON metabolite α-ZOL has been documented as having greater potency than ZON or ß-ZOL based on tissue binding studies (9), and *in vitro* assays (15, 42). Swine are thought to be most sensitive to ZON, at least in part, due to the higher metabolic production of α-ZOL vs. ß-ZOL (16) than found for chickens, sheep, cattle or rats (15). Yet swine studies have often been performed at higher doses per kg of body weight (BW) than studies in other species. When multiparious sows were fed up to 100 ppm or 1.25 mg/kg BW, reproductive effects were obvious: pseudopregnancy, mammary gland ductal hyperplasia, and thickened uterine and vaginal epithelium (43). Dosing prepubertal gilts with 192 μg ZON/kg BW/da for 4 days, also resulted in anestrus and swollen vulvas (16). In our study on horses, 8 mg ZON/da fed to a 450 kg mare equates to approximately 18 μg/kg BW/da rather than a dose on the order of mg/kg BW/da as tested in swine.

Some would ignore the dose realities discussed above, and would maintain that lower sensitivity to ZON in horses is due to (1) production of the less active ZON metabolite (ß-ZOL) vs. α-ZOL, and (2) increased metabolism and subsequent excretion of ZON (35). Adaptation of various physiological parameters to ZON exposure was also reported in chronic exposure of prepubertal gilts (44). While metabolic parameters and hormone profiles may not indicate effects of ZON with chronic exposure, fertility in horses may be negatively impacted by even a 2 mg/da dose. It is possible that, as has been found with induction of carcinogen metabolizing hormones (45, 46), there is a concentration where an exogenous compound elicits the same response in an animal as an endogenous or “natural” compound, and therefore the animal fails to mount a compensatory response. However, when exposed to the same compound at higher concentrations, it induces feedback systems supporting homeostasis (for example P450 induction) in order to reestablish a “normal” state. Chronic exposure of mares to ZON at 2–8 mg/da resulted in no statistical effect on examined hormone profiles, and no obvious toxicological effects, though the potential for a decrease in the number of foals produced/mare cannot be completely ruled out.

## Data Availability Statement

The datasets generated for this study are available on request to the corresponding author.

## Ethics Statement

This animal study was reviewed and approved by Mississippi State University Institutional Animal Care and Use and Committee, protocol #12-022.

## Author Contributions

PR, NS, and EK conceived and designed the study. EK, SB, KW, and PR collected clinical data and blood samples. SB and NS conducted the hormone and estrogenicity assays. CV, EK, SB, and NS organized and interpreted the database. CV, EK, and NS analyzed the data and performed the statistical analysis. CV and NS wrote and revised the manuscript. All authors contributed to the manuscript, read, and approved the submitted version.

### Conflict of Interest

The authors declare that the research was conducted in the absence of any commercial or financial relationships that could be construed as a potential conflict of interest.
