# Supplementary material for: Reproductive Performance of Mares Fed Dietary Zearalenone
Source: Front Vet Sci. 2019 Nov 26;6:423. doi: 10.3389/fvets.2019.00423 (PMC6988787; doi:10.3389/fvets.2019.00423)
Supplement: Supplementary file 1 [file Table_1.DOCX]

Table 1. Summary of Kenney-Doig endometrial biopsy categories and prognoses for uterine biopsies in mares^a,b^

| Category | Major Findings | Predicted Foaling Rate (%) |
| --- | --- | --- |
| I | Essentially normal, inflammation or fibrosis slight and sparsely scattered | 80-90 |
| IIA | Mild, scattered inflammation; mild fibrosis*; endometrial atrophy in late breeding season | 50-80 |
| IIB | Moderate, scattered inflammation; moderate fibrosis* | 10-50 |
| III | Severe, irreversible changes including fibrosis and inflammation | 10 |

*Qualifying lesions additive in nature; more than one qualifying lesion increases the category

^a^ Snider,T., Sepoy, C., Holyoak, G. 2011. Equine endometrial biopsy reviewed: Observation, interpretation, and application of histopathologic data. *Theriogenology* 75, 1567-1581.

^b^Kennedy, R. M. and Doig, P.A. 1986. Equine endometrial biopsy, Current therapy in theriogenology 2. Philadelphia: WB Saunders, 723-9.
