# Supplementary figures and images for: Reproductive Performance of Mares Fed Dietary Zearalenone
Source: Front Vet Sci. 2019 Nov 26;6:423. doi: 10.3389/fvets.2019.00423 (PMC6988787; doi:10.3389/fvets.2019.00423)

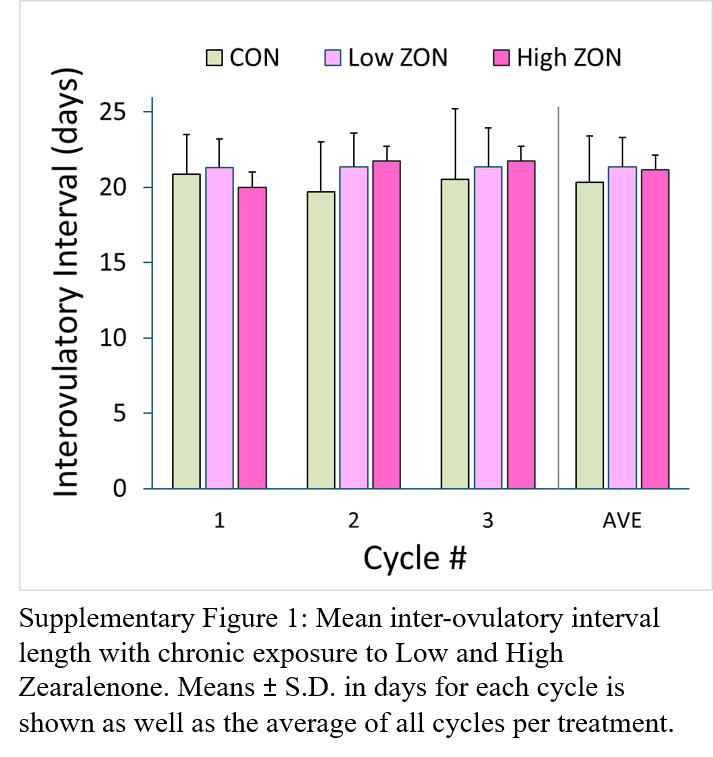

Supplement: Supplementary file 2 [file Image_1.TIF]

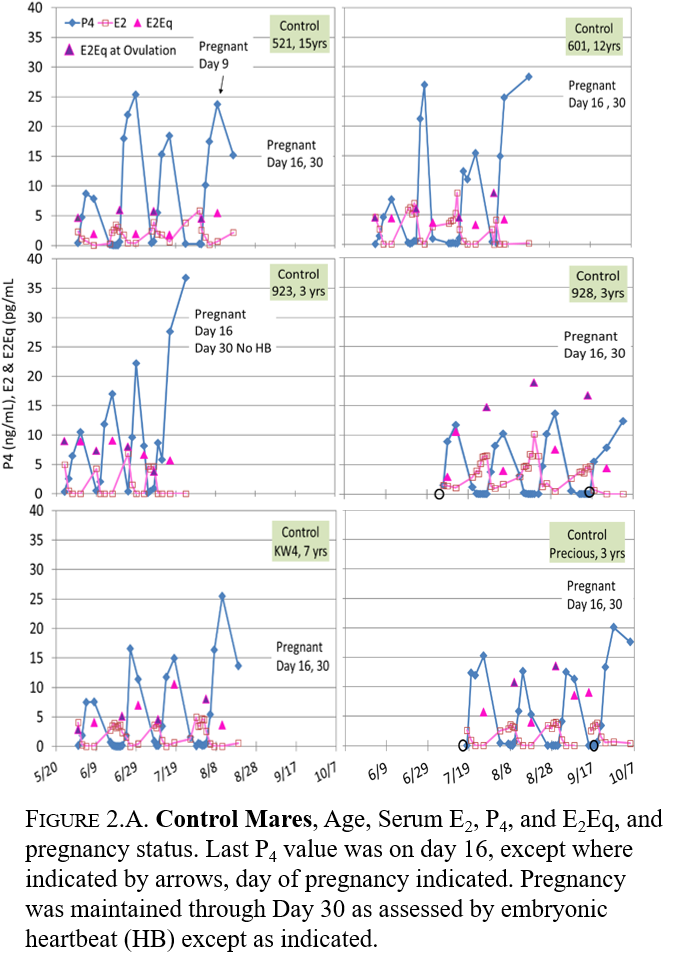

Supplement: Supplementary file 3 [file Image_2.TIF]

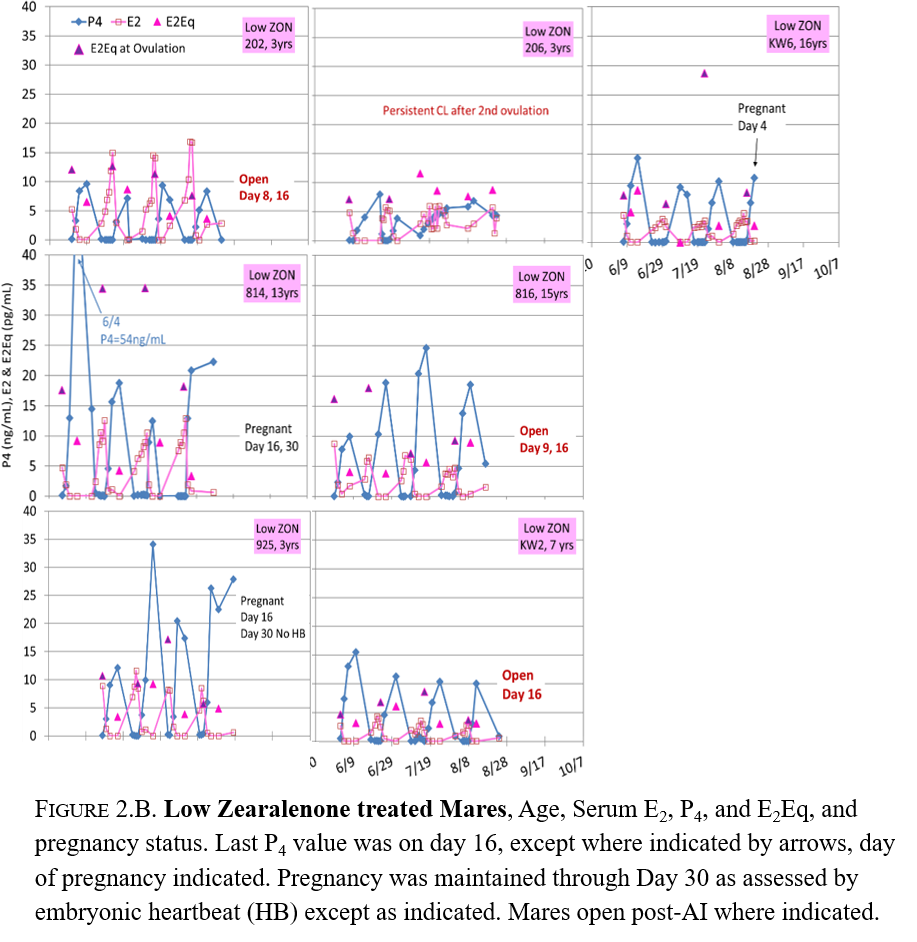

Supplement: Supplementary file 4 [file Image_3.TIF]

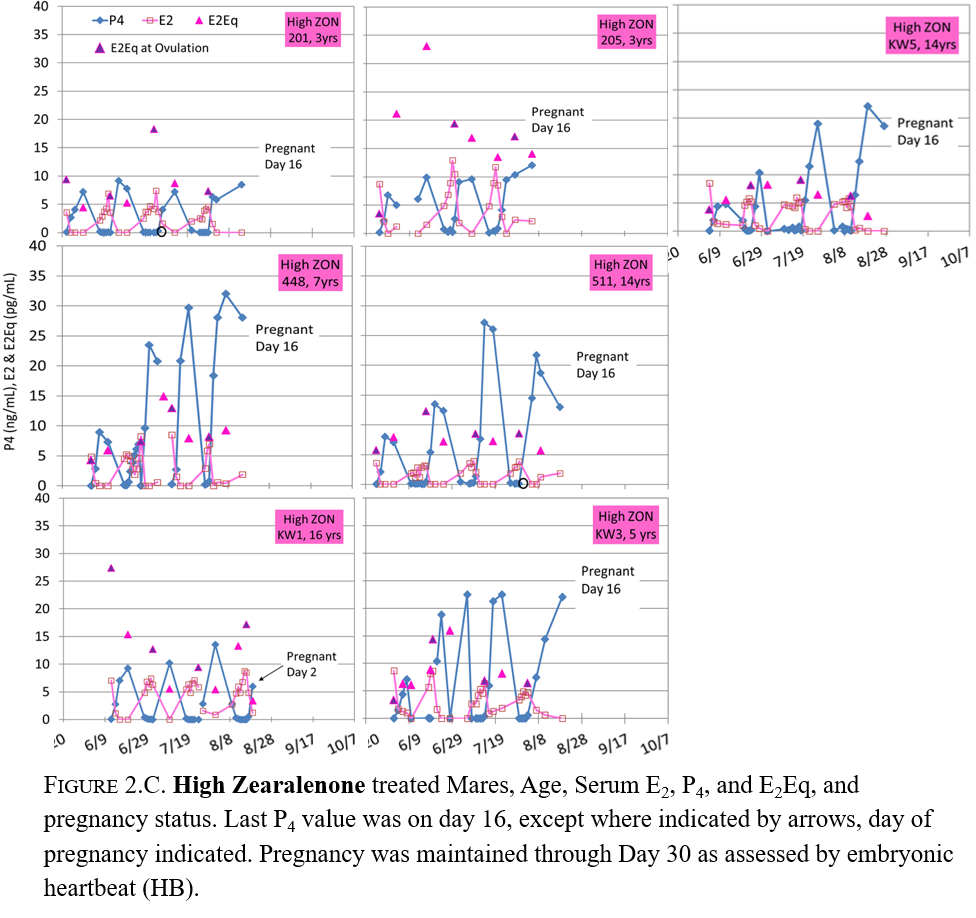

Supplement: Supplementary file 5 [file Image_4.TIF]
